# Two different mechanisms alternate during cortical synchronized states

**DOI:** 10.1186/1471-2202-16-S1-P264

**Published:** 2015-12-18

**Authors:** Erin Munro, Tansi Khodai, Shuzo Sakata, Taro Toyoizumi

**Affiliations:** 1 Brain Science Institute, RIKEN, Wakoshi, 351-0198, Japan; 2 Centre for Neuroscience, University of Strathclyde, Glasgow, G4 ORE, UK

## 

Brain states can be classified as synchronized (large amplitude low frequency oscillations) or desynchronized (small amplitude high frequency activity). [1] Synchronized states are marked by UP states/phases characterized by global spiking and DOWN states/phases are characterized by global silence in the cortex. In awake animals, desynchronized states are associated with processing sensory input and behavior while synchronized states are associated with quiet idling conditions. During sleep, REM is considered desynchronized and slow-wave sleep is considered synchronized. While desynchronized brain states are often triggered by various kinds of neuronal input to cortical areas, the exact mechanism at work during synchronized brain states is still unclear. In particular, there are two hypothesized mechanisms for the slow oscillation during slow-wave sleep: UP phases can be produced either by traveling neocortical waves or a thalamo-cortical loop [2-4].

In our study, applying independent component analysis (ICA) to recordings from rat neocortex reveals two different mechanisms during synchronized activity. The mechanisms are distinguished by two key neural sources identified by ICA: a strong broad source centered in layer 5 (BL5) and an apparent sub-cortical source producing clock-like oscillations which resemble hippocampal theta oscillations (SUB). The BL5-state often resembles cortically generated oscillations: UP phases are initiated in deeper layers akin to traveling neocortical waves and the oscillation is relatively slow. The SUB-state can resemble thalamo-cortically generated oscillations: UP phases are initiated in layer 4 as well as deeper layers and the oscillation is faster. These findings suggest that both hypothesized mechanisms for the slow oscillation are at work in the cortex - in alternation.
